# An attempt to valorize the only black meat chicken breed of India by delineating superior functional attributes of its meat

**DOI:** 10.1038/s41598-022-07575-9

**Published:** 2022-03-03

**Authors:** Rekha Sharma, Renuka Sehrawat, Sonika Ahlawat, Vivek Sharma, Alka Parmar, M. S. Thakur, A. K. Mishra, M. S. Tantia

**Affiliations:** 1 ICAR-National Bureau of Animal Genetic Resources, Karnal, 132 001 India; 2 ICAR- National Dairy Research Institute, Karnal, 132 001 India; 3 Nanaji Deshmukh Veterinary Science University, Jabalpur, 482001 India

**Keywords:** Animal biotechnology, Biotechnology, Biochemistry, Peptides

## Abstract

Kadaknath, the only black chicken indigenous to India, faces the threat of extinction due to declining numbers. Its meat is used in tribal medicine for invigorating and health-promoting properties. Expectations of immune-boosting and therapeutic properties in its meat are creating a buzz these days. Thus, Kadaknath meat was explored and further compared with the commercial Cobb 400 broiler (Cobb) for the functional traits that might be contributing towards proclaimed pharmacological benefits. Birds (n = 20/ group) were raised under similar management conditions and the two primal chicken meat cuts (breast and thigh) were collected at the marketing age. Kadaknath meat was found to be an enriched source of functional biomolecules (carnosine, anserine, creatine). Its breast meat carnosine content was more than double of the Cobb broiler, 6.10 ± 0.13 and 2.73 ± 0.1 mg/ g of wet tissue, respectively. Similarly, the thigh meat of Kadaknath was a significantly (P < 0.05) richer source of carnosine. The genetic background was a key determinant for muscle carnosine content as a significant abundance of CARNS1 and SLC36A1 expression was identified in the Kadaknath breast. The superior functional property of Kadaknath meat was established by the antioxidant capacity established by the Oxygen radical absorbance capacity assay and a stronger ability to inhibit the formation of advanced glycation end products (AGEs). The identification of fairly unknown nutritional and functional advantages of Kadaknath meat could potentially change the paradigm with its meat consumption. It will help in developing a brand name for Kadaknath products that will propel an increase in its market share and ultimately conservation of this unique but endangered poultry germplasm.

## Introduction

Healthy eating is being emphasized amid the Corona pandemic crisis for boosting natural immunity^1^. Several nutrients have health-promoting effects that have clinical utility in preventing or managing COVID-19 (e.g. vitamins B, C, and D) and several others are attracting attention for their potential use, such as histidine-containing dipeptides (HCDs)^2,3^. Knowledge about the numerous ergogenic and therapeutic properties of HCDs-carnosine (N-β-alanyl-l-histidine) and anserine (N-β-alanyl-1-methyl-l-histidine), is widespread in the scientific literature^4,5^. These can counteract the development of several chronic diseases due to their role as antioxidants, antiglycation, and anti-inflammatory agents^6^. Dietary intake of HCDs promotes the immunological defense of humans against infections by bacteria, fungi, parasites, and viruses (including corona virus) by enhancing the metabolism and functions of monocytes, macrophages, and other cells of the immune system^7^. Carnosine, anserine, and creatine have been considered bioactive compounds for human consumption due to the crucial role performed in shielding the mammalian cells from oxidative damage^8^. These are copiously found in meat and are absent in vegetarian foods^9^. Poultry meat has the highest total HCD content among the farm animal species^10,11^. Consequently, chicken meat has the highest antioxidant capacity in comparison to pork, beef, and fish^12,13^. Poultry meat is a good source of natural dipeptide carnosine a nutritional supplement that could ameliorate some of the damaging effects of SARS-CoV-2 infection^1^. It has been established to have a potential role in the treatment of mice infected with swine flu H9N2 and inhibition of Zika and dengue virus infection as well as replication in human liver cells^14^. Lopachev et al.^15^ proposed salicyl-carnosine as a promising candidate drug for the treatment of patients with severe cases of COVID-19 infection.

**Figure 1:**
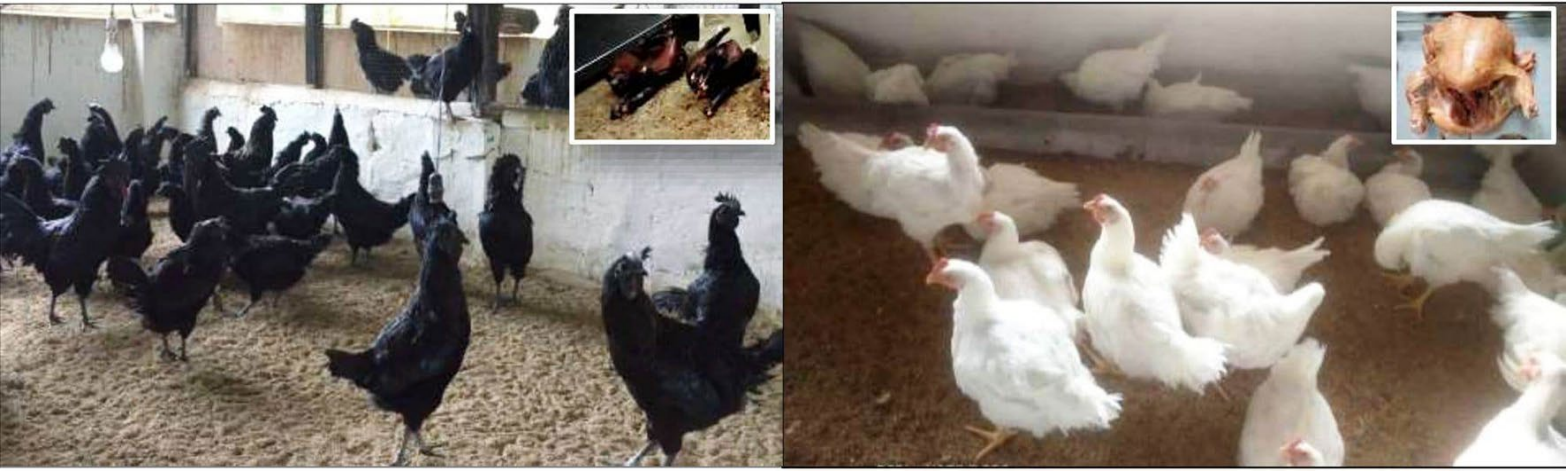
Representative flocks of the Kadaknath chicken (black) and Cobb 400 broiler (white) with the respective carcass in the inset.

Variation in the HCD content has been reported among different chicken genotypes^16^. Black meat of Chinese Silky fowl that is used in traditional Chinese medicine had a higher carnosine level than the commercial broiler (White Plymouth Rock) and five other poultry breeds^17,18^. All-black chicken breeds include Silkie in China, Ayam cemani in Indonesia, and Kadaknath in India. The autochthonous Kadaknath is the only black meat chicken breed (Accession No. INDIA_CHICKEN_1000_KADAKNATH_12009) among the 19 chicken breeds registered in India (https://nbagr.icar.gov.in/en/home/)^19^. The tribal community of *Bhil* and *Bhilala* are the primary custodian of this unique backyard poultry. It is the only indigenous animal genetic resource in India to get the Geographical Indication (GI) tag for the protein-rich and black-colored meat in 2018 (https://ipindia.gov.in/writereaddata/Portal/IPOJournal/1_2598_1/Journal_104.pdf). The peculiarity of this breed is that the entire bird and its internal organs are black (Fig. 1) due to the deposition of melanin pigment, a genetic condition called "Fibromelanosis"^19^. It is supposed to have aphrodisiac and medicinal properties and is used in the treatment of many human diseases^20,21^. Consequently, it holds a special place in the livelihood of the tribal populations^22^. However, its population is rapidly declining due to the lower production potential and hence it is under the threat of extinction as well as genetic erosion.

Commercialization of intensive broiler chicken production across the globe is resulting in decreased poultry genetic diversity around the world^23^. This trend is particularly damaging to backyard small-scale poultry rearing that supports food and nutritional security and poverty alleviation in developing countries including India^24^. Currently, the focus has shifted to healthy and natural foods that resulted in a renewed interest in native chickens^25^. The demand for black meat of Kadaknath has increased amidst the ongoing Corona pandemic due to the expectations of the improved immunity status of human beings (https://icar.org.in/node/8075). But, there is a paucity of literature that can endorse the claims of nutritional and medicinal properties of Kadaknath meat. Scientific knowledge on the nutritional constituents that contributes towards the functional characteristics of its meat can impart a brand name to this unique poultry. It will open the door for exploiting the market potential of consumers interested in nutritional and therapeutic quality, environmental sustainability, and animal welfare, which in turn, will also support Kadaknath breed conservation.

Hence, the present study was aimed to determine and compare the HCDs (carnosine and anserine) concentration, expression of related enzymes and transporters as well as the antioxidative and antiglycation potential of the Kadaknath chicken meat (breast and thigh) with the commercial Cobb 400 broiler (Cobb). It was hypothesized that the Kadaknath chicken meat has an edge over the commercial broiler meat for the targeted nutritional properties.

## Results

The possible supremacy of Kadaknath chicken over the commercial Cobb broiler was explored based on the concentration of carnosine and its related physiological activity in the meat. Breast and thigh meat as a typical representative of the white and red muscles were also compared among the two groups of chicken.

### Kadaknath meat is enriched in carnosine

**Figure 2:**
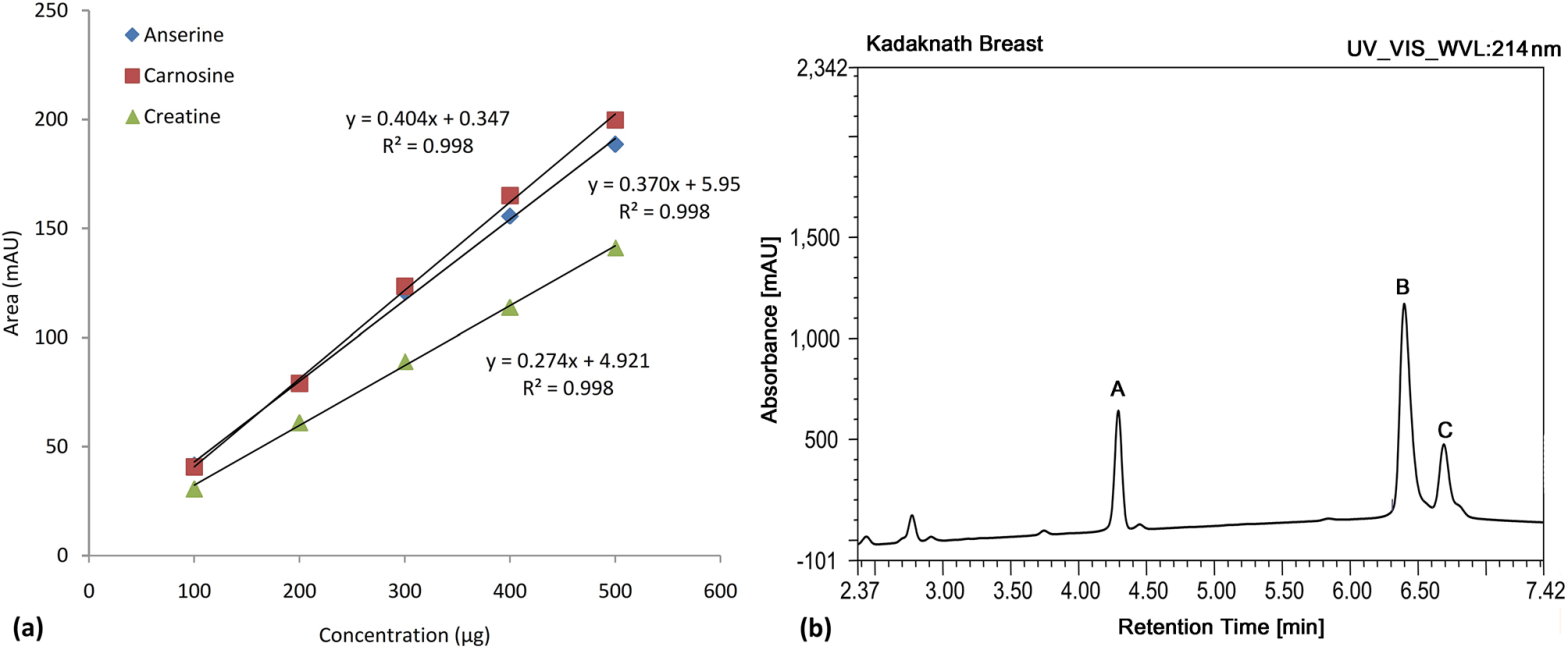
(**a**) Linearity range and regression of anserine, carnosine, and creatine standard solutions (100–500 µg) separated by high-performance liquid chromatography (HPLC) (**b**) A representative chromatogram identifying creatine (A), anserine (B), and carnosine (C) in a meat sample at 214 nm.

The concentration of selected functional molecules; HCDs (carnosine and anserine) was determined using the HPLC. In addition, another functional molecule creatine [*N*-(aminoiminomethyl)-*N*-methyl-glycine], that plays a vital role in the energy metabolism of skeletal muscle was estimated simultaneously. A standard curve of varying concentrations was prepared for pure carnosine, anserine, and creatine for the calculation of respective components in the meat samples (Fig. 2a). The R^2^ obtained for the regression line was 0.998. All three metabolites were separated within 8 min and a typical chromatogram is presented in Fig. 2b.

A considerable amount of all the three targeted molecules were detected in the breast and thigh meat extracts (Table 1). Carnosine content was significantly different between the Kadaknath and Cobb groups as well as among the two types of meat cuts; breast and thigh. The average carnosine concentration (mg/g of tissue) in Kadaknath breast (6.10 ± 0.13) and thigh (1.71 ± 0.10) was more or less twice the corresponding values in Cobb broiler. Breast tissue has a higher carnosine concentration than the thigh and the difference was more than twofold in the Cobb broiler and more than threefold in the Kadaknath. Similarly, anserine concentration was significantly higher in the breast tissue as compared to the thigh tissue of Kadaknath as well as, the Cobb broiler. However, unlike carnosine, the concentration of anserine among the two poultry groups did not differ significantly. Anserine was the major HCD in the meat of Cobb broiler (4.85 ± 0.22 vs 2.73 ± 0.10 carnosine, mg/ g of tissue), whereas carnosine content (6.10 ± 0.13) was higher than the anserine (5.0 ± 0.14) in the Kadaknath chicken. A similar concentration of creatine was quantified across the two poultry groups, irrespective of the type of tissue (Table 1). However, among the breast and thigh meat, the breast meat had higher HCD concentrations.

**Table 1:** Histidine containing dipeptides (HCDs), and creatine concentration in the chicken meat.

Metabolite (mg/g of tissue)	Genotype (n = 20)	Breast		Thigh	
		Mean ± SEM	Range	Mean ± SEM	Range
Carnosine	Cobb broiler	2.73 ± 0.10^a^	2.11–3.39	0.98 ± 0.03^a^**	0.78–1.20
	Kadaknath chicken	6.10 ± 0.13^b^	5.46–7.11	1.71 ± 0.10^b^**	1.14–2.38
Anserine	Cobb broiler	4.85 ± 0.22^a^	3.51–6.33	2.27 ± 0.14^a^**	1.67–3.14
	Kadaknath chicken	5.0 ± 0.14^a^	4.0–6.06	1.88 ± 0.12^a^**	1.33–2.80
Creatine	Cobb broiler	3.06 ± 0.12^a^	2.39–3.86	2.56 ± 0.10^a^**	2.0–3.25
	Kadaknath chicken	2.92 ± 0.17^a^	2.22–3.60	2.48 ± 0.13^a^**	1.74–3.35

Values are mean ± SEM (n = 20). Means with different letters in the same column and different superscripts within the same row are different (P < 0.05).

### Expression of the carnosine-related genes

The expression of genes for the transporters and enzymes involved in the muscle cell homeostasis of carnosine was investigated. These included carnosine metabolism-related enzymes; Carnosine synthase1 (CARNS1), Carnosine dipeptidase (CNDP1 and CNDP2), and transporters; solute carrier family 6, member 6 (SLC6A6), solute carrier family 6, member 14 (SLC6A14) and solute carrier family 36, member 1 (SLC36A1). Kadaknath and Cobb broiler were compared using the breast meat due to the better carnosine concentration (Table 1). The molecular basis of difference in the breast and thigh meat was elucidated using the carnosine enriched Kadaknath meat.

Preliminary qRT-PCR assays were performed for the six selected genes and it was observed that CARNS1, CNDP2, SLC6A6, SLC36A1 genes were expressed in both the breast and thigh tissues. However, CNDP1 and SLC6A14 genes did not show any expression at the limits of detection for employed qRT-PCR.

**Figure 3:**
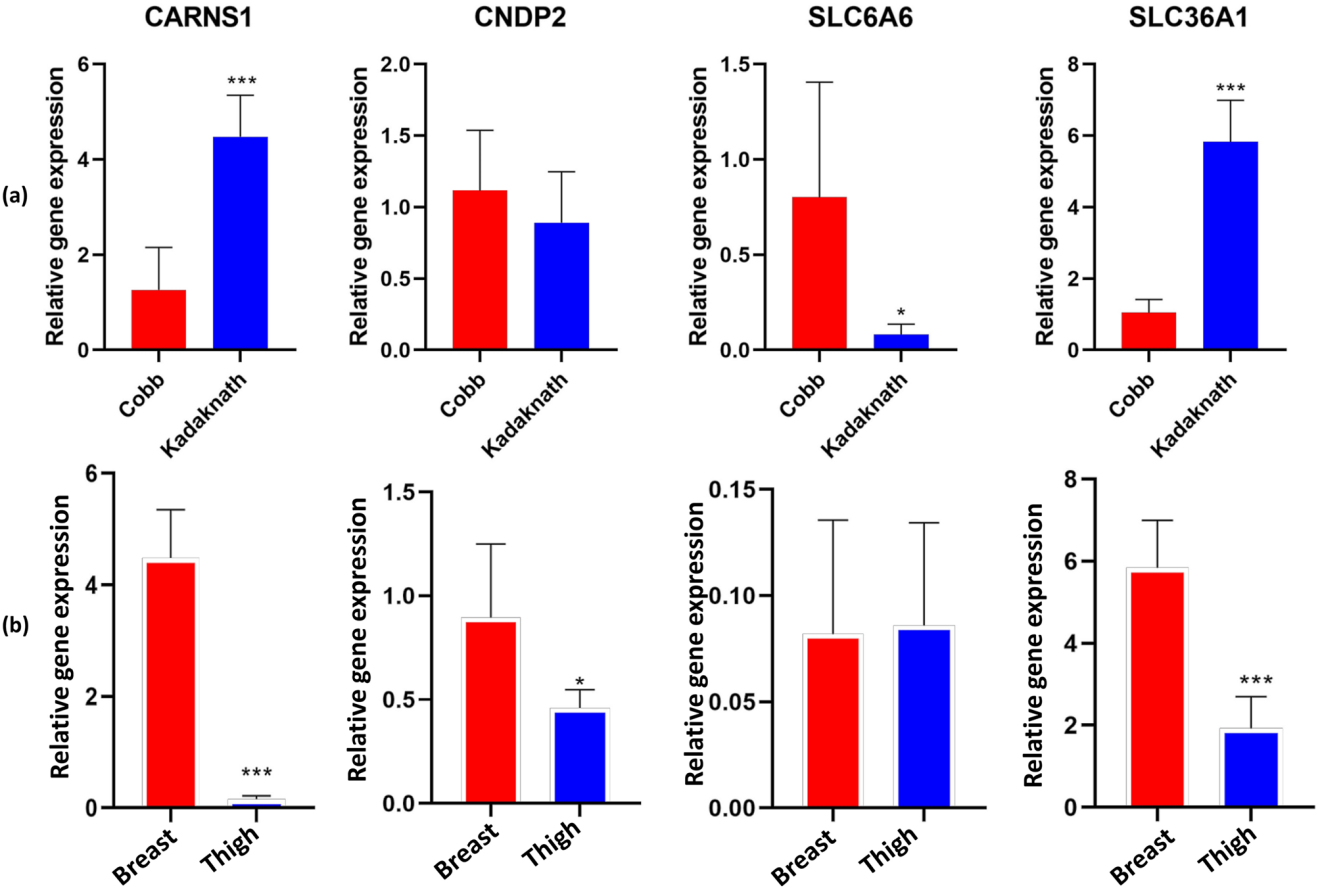
Relative mRNA abundance of CARNS1, CNDP2, SLC6A6, and SLC36A1 (**a**) Cobb broiler Vs Kadaknath breast (**b**) breast Vs thigh of Kadaknath. Error bars represent the standard deviations from triplicate qRT-PCR runs. (***P < 0.001, *P < 0.05).

Comparative gene expression profile of Kadaknath and Cobb breast meat (Fig. 3a) revealed that mRNA abundance of CARNS1 was higher (P < 0.001) in the Kadaknath as compared to the Cobb broiler. Whereas, a significant difference was not observed among the two groups for the transcripts of CNDP2. The β-alanine transporter genes SLC6A6, as well as SLC36A1, were also differentially expressed. The mRNA abundance of SLC36A1 was higher (P < 0.001) and that of the SLC6A6 gene was lower (P < 0.05) in the Kadaknath breast as compared to the Cobb broiler. However, the magnitude of difference for an increase in SLC36A1 (fold change) outnumbered the decrease of SLC6A6 expression in the Kadaknath meat. Collectively, Kadaknath breast tissue has a several-fold higher expression for both the enzyme (CARNS1) and transporter (SLC36A1) contributing towards the enhanced synthesis of carnosine than the Cobb broiler.

Comparative expression of carnosine-related genes in the breast and thigh meat indicates that the breast has several-fold higher expression of enzymes and transporter (SLC36A1, CARNS1, CNDP2) that drive increased carnosine accumulation (Fig. 3b). Similarly, the expression of CNDP2 was also significantly higher in the breast. The difference observed in the transcript abundance among the two types of meats was in agreement with the results obtained for carnosine concentration in breast and thigh tissues (Table 1).

### Functional properties of meat

**Figure 4:**
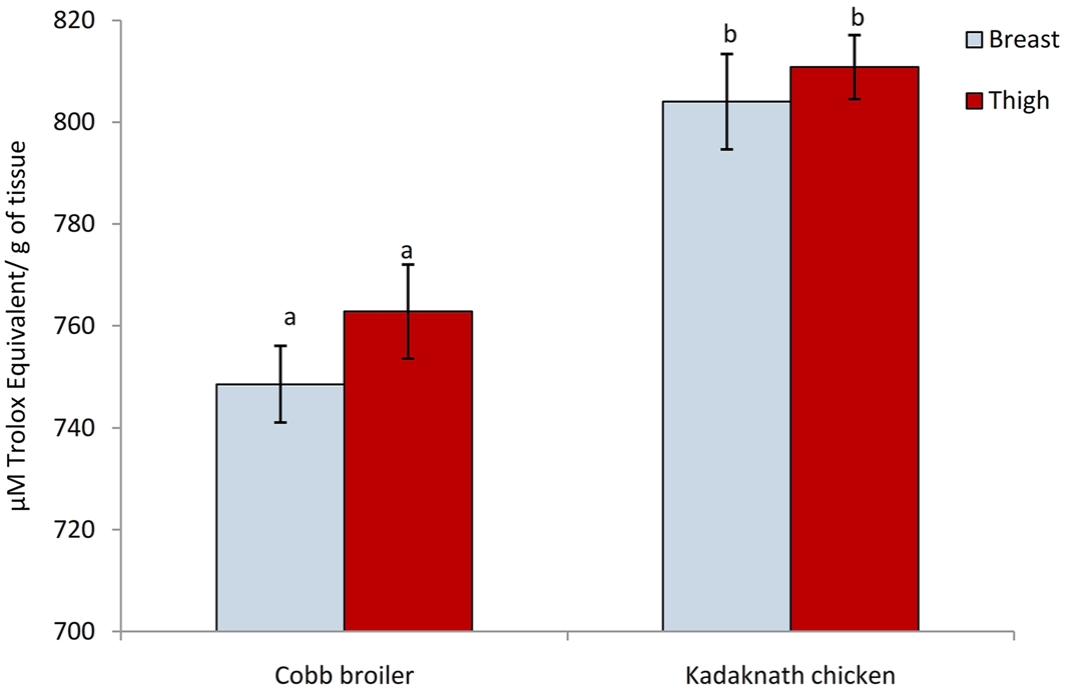
Antioxidant capacity as estimated by Oxygen radical absorbance capacity (ORAC) assay. Results are expressed as means with an error bar indicating the standard error of the means (n = 20). Different letters on the bars indicate significant differences (P < 0.05).

Antioxidative capacity gives a valuable indication of the functional property of meat. Thus, the total antioxidant capacity was evaluated using an Oxygen radical absorbance capacity (ORAC) assay for a comparison between Kadaknath and Cobb meat antioxidant capacities. This method uses an area-under-curve (AUC) technique and thus combines both inhibition time and inhibition degree of free radical action by an antioxidant. Results (Fig. 4) showed the higher antioxidant capacity of Kadaknath breast and thigh meat over that of the Cobb broiler. The Trolox equivalent antioxidant capacity (TEAC) of Kadaknath breast and thigh meat was calculated to be 804.01 ± 9.37 and 810.8 ± 6.29 (µM Trolox equivalent (TE)/g of tissue), respectively while corresponding values were 748.56 ± 7.48 and 762.82 ± 9.19 (µM Trolox equivalent (TE)/ g of tissue), in the meat of Cobb broiler. There was no significant difference among the breast and thigh tissue of the same genetic group.

**Figure 5:**
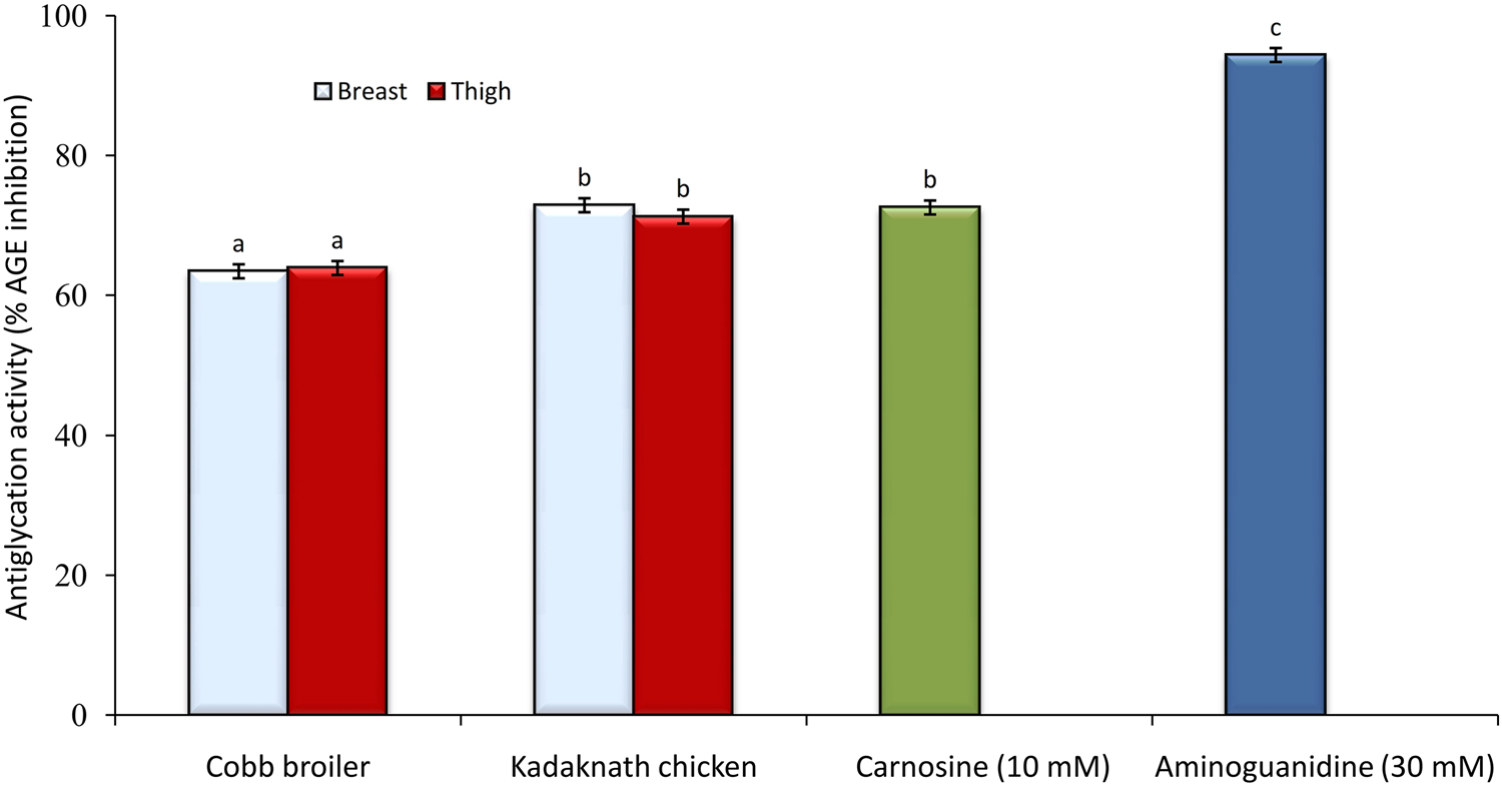
Antiglycation effects of meat extracts in the bovine serum albumin-methylglyoxal model. Aminoguanidine (30 mM) served as the positive control. Bars represent mean ± standard error (n = 20). Bars with different letters denoted significant differences (P < 0.05).

A comparative study between Kadaknath and Cobb broiler was accomplished to evaluate the ability of their meat extract to inhibit the formation of advanced glycation end-products (AGEs). To measure the antiglycation ability, the bovine serum albumin–methylglyoxal (BSA-MGO) system was used as the marker of the middle stage of the formation of oxidative cleavage products. Kadaknath and Cobb broiler meat extracts were able to inhibit the formation of fluorescent AGEs in the BSA-MGO model (Fig. 5). Kadaknath breast meat presented a better anti-glycation potential (72.89 ± 0.81% AGEs inhibition) compared to the Cobb broiler (63.43 ± 0.89% AGEs inhibition). Similarly, thigh meat extract of Kadaknath and Cobb broiler inhibited fluorescent AGEs generation by 71.25 ± 1.21% and 63.91 ± 0.98%, respectively. There was no significant difference among the breast and thigh tissue of the same group. The antiglycation activity of Kadaknath meat was similar to that of pure carnosine (72.57 ± 0.32% AGEs inhibition).

## Discussion

Carnosine concentration in Cobb broiler meat (Table 1) corroborated with the previously described values in the broiler meat. Maikhunthod and Intarapichet^26^ reported carnosine levels of 2.9 mg/g for broiler chicken breast muscle and 0.49 mg/g for the thigh, and Mori et al.^27^ reported carnosine concentration of 2.55 ± 0.26 mg/g for chicken breast and 1.06 ± 0.18 mg/g for the thigh meat. Korean native chicken (KNC) carnosine concentration was also of similar magnitude^16,28^. On the other hand, carnosine concentration in both the breast and the thigh meat of Kadaknath was approximately twice the Cobb broiler (Table 1). Similarly, black chicken of China, Silky fowl (SF) had 1.7- to 1.9-fold higher carnosine in comparison to the four chicken varieties, Nagoya Breed (NB), Japanese Game Cross (JG), Hinai-jidori (HJ), and commercial broiler^18^. SF breast meat had a carnosine concentration of 7.98 ± 0.86 mg/g meat, that was highest in comparison to the other varieties, JG (4.55 ± 0.37 mg/g), HJ (4.41 ± 0.5 mg/g), and NB (4.79 ± 0.24 mg/g). Similarly, maximum thigh meat carnosine (2.88 mg/g) was quantified in the SF black chicken^18^. Concurrent to present findings, SF thigh meat had 1.6- to 2.3 fold higher carnosine levels. Previously, Tian et al.^17^, reported remarkably higher carnosine in the SF mixed meat, breast meat, and thigh meat in comparison to the White Plymouth Rock, bred under the same condition.

Anserine is the main HCD in the meat of poultry and salmonid fishes, whereas carnosine is the principal HCD in pork and beef^13,28^. Their ratio varies according to the species of animal and type of muscle^29^. Anserine concentration in the meat of chicken and rabbit was more than twice the carnosine concentration^10^. This trend was also followed in the native chicken of Korea and was not affected either by the age of the chicken, the type of meat, or the status of the meat^16^. Similarly, anserine is the major HCD in skeletal muscles of commercial broilers^18^, including Ross 308^30^ and the same was the case with the Cobb broiler in the present study (Table 1). Surprisingly, this trend was not observed in the Kadaknath breast meat as it had higher carnosine content (6.10 ± 0.13 mg/ g of tissue) than the anserine (5.0 ± 0.14 mg/ g of tissue). The only other report on a higher carnosine rather than the anserine in breast meat originates from the SF^18^. Thus, a predominance of carnosine instead of anserine can be considered a noteworthy characteristic of black chicken meat. Among the two types of meat cuts, anserine concentration was significantly higher in the breast tissue of both the chicken groups (Table 1). Higher anserine concentration in the chicken breast meat as compared to the thigh has been reported for broiler, KNC, and black SF chicken^16,18^.

Many factors may affect the carnosine concentration in meat like genetic factors, rearing conditions, type of muscle fiber, gender, age, etc.^5^. Therefore, management conditions were kept identical, only male birds were selected, and sample collection, processing, and analysis were done simultaneously to minimize the variables. Birds were selected for comparison at their market age (Cobb broilers—8 weeks, Kadaknath—20 weeks) to compare the quality of meat that is consumed. Hence, Kadaknath meat was potentially matured as compared to Cobb. However, age may not be the reason for better HCDs in the Kadaknath as it has earlier been reported that the carnosine and anserine content of the breast and thigh meat (raw or cooked) did not increase significantly with the age of KNC^16^. Similarly, Jung et al.^28^ did not find any correlation between the body weight and content of anserine in the meat of five different lines of KNC. Moreover, the amount of type IIb fibers remained unchanged among fast-growing and slow-growing chicken types^31^. Consequently, it can be contemplated that the better carnosine in the Kadaknath meat may correspond to some specific important physiological role in its muscles. For instance, animals that need to escape from predators such as greyhound dogs have a higher percentage of fast-twitch glycolytic fibers and hence need more HCDs for efficient buffering^32^. The same may be true for the Kadaknath, a free-range bird in need of protection from predators. The Kadaknath chicken has a slower growth rate that might be reducing the demand for amino acids required for synthesizing protein in tissues. As a consequence, dietary amino acids may be spared for synthesizing non-proteinogenic biological compounds such as HCDs. In both the chicken groups carnosine and anserine were more than double in the breast than the thigh (Table 1), indicating that the HCDs concentration was affected by the muscle allocation. Characteristics of muscle fiber were not evaluated in the current study but recently, a three-fold higher concentration of HCDs in the breast compared to the thigh muscles of Ross birds was allocated to the myofiber allocation^30^. Published literature also supports higher HCDs in the chicken breast muscle than the thigh muscle^26,29,30,33^. Similarly, breast meat had a better creatine concentration than thigh meat (Table 1). Creatine and creatine phosphate plays an imperative role in the energy metabolism of muscle and supply the energy obligatory for the forceful muscle contraction^34^. Phosphocreatine is a prerequisite for instant ATP regeneration and correlates with the higher level of creatine in glycolytic muscles of the breast^34,35^.

Breast and thigh muscles can be considered as typical representatives of white and red muscles. A higher concentration of carnosine has been found in the white muscle rather than the tissue having an abundance of red muscle^36^. The fast-twitch type IIb (glycolytic) fiber predominate the chicken breast, whereas the thigh is mostly composed of type I oxidative fiber^26^. Type IIb fibers in the breast muscles are involved in short bursts of muscular activity requiring fast contraction. Moreover, restrictive oxygen supply exists due to the low myoglobin and limited capillary network^28^. Thus, ATP is generated through anaerobic fermentation of glucose to lactic acid via the glycolytic pathway and results in an acidic environment inside the cell^31^. This may be the reason for the need for a higher quantity of dipeptides to act as a physio-chemical buffer against protons^37,38^. Whereas, type I fibers contract slowly and are highly resistant to fatigue^26^. They have a better capillary supply, have high myoglobin content, and thus perform aerobic metabolism. This may elucidate the reason for lower priority for thigh HCDs synthesis in comparison to muscles of the breast.

Expression of transporters and enzymes related to the carnosine accumulation explained the molecular basis for the increased carnosine build-up in the Kadaknath black meat. Everaert et al.^39^ also proposed that variation in the carnosine concentration among chicken breeds may be due to genotypic differences. Synthesis and hydrolysis are the primary pathways involved in carnosine metabolism, from and to its constituent amino acids, respectively^40^. CARNS1 is characterized as an ATP-dependent cytosolic enzyme, which catalyzes the carnosine synthesis from amino acids β-alanine and L-histidine^5^. It is mostly expressed in skeletal muscles^27^, some regions of the brain such as the olfactory bulb, and in the heart^42^. Carnosine is hydrolyzed in tissues including skeletal muscles by a cytosolic Zn^2+^-dependent carnosinase-2 (CNDP2) enzyme. Expression of CARNS1 was higher in the Kadaknath meat as compared to the Cobb broiler (P < 0.001). Concurrent to the present study, CARNS1 and CNDP2 have been reported in the skeletal muscle of humans, pigs, and mice^41^. Our results were in agreement with previous reports in the pig skeletal muscles where CARNS1 gene expression and transcripts corresponded to the muscle carnosine content^41^. β-alanine is thought to be the rate-limiting precursor of carnosine synthesis in humans and horses^42^. Expression of SLC6A6, as well as SLC36A1, has been reported in mouse, pig, and human skeletal muscles^41,43^. SLC6A6 transporter is a Na^+^ and Cl^−^ coupled transporter that can transfer both taurine and β-alanine^43^. The SLC36A1 gene corresponds to a high-capacity, low-affinity transporter for various amino acids including taurine and β-alanine. If the SLC6A6 gets saturated in the tissues then the SLC36A1 might permit the mass substrate transfer^44^. Thus, several-fold higher expression of SLC36A1 in the Kadaknath compared to the Cobb broiler may account for the availability of abundant β-alanine inside the muscle cells. Collectively, Kadaknath breast tissue has several-fold higher expression than the Cobb broiler for both the enzyme (CARNS1) and transporter (SLC36A1) that may be driving the enhanced carnosine synthesis.

A higher concentration of carnosine in the breast meat as compared to the thigh meat was also in line with the expression profile of related genes (Fig. 3). Although the expression of CNDP2 was also higher in the breast, the effect was offset by the much higher magnitude of the CARNS1 and SLC36A1 expression. Moreover, degradation by the corresponding carnosinase-2 is very limited in the muscle cell due to the lower pH (7.1) than the optimal pH (9.5) of this enzyme^45^. Literature is naïve in explaining the molecular basis of carnosine differences across the two types of chicken muscles. Limited information exists for other species. D’Astous-Page et al.^41^ observed the highest value of mRNA transcript of CARNS1 in pig breast muscle (*longissimus* *thoracis)* as compared to duodenum, kidney, lung, and backfat. Similarly, the maximum mRNA levels were reported in the human glycolytic muscles (*gastrocnemius* and *tibialis* *anterior*) than that in the oxidative muscles of the heart (*soleus*)^42^.

The absence of CNDP1 and SLC6A14 genes expression in the present study was not surprising. The lack of transcript for CNDP1 was justifiable as this peptidase was acknowledged to be the carnosinase of serum that is expressed primarily in the rodent kidney, human brain tissues, and liver^41,46^. CNDP1 was undetectable in muscles of the mouse (*tibialis* *anterior*), human (*gastrocnemius*), and pig (l*ongissimus* *thoracis*)^36,39^. Similarly, β-alanine transporter SLC6A14 was not expressed in human (*gastrocnemius*) and mouse (*tibialis* *anterior*) skeletal muscle^39^. Recently, Qi et al.^43^ reported that the CNDP1 and SLC6A14 were not detected in the chicken breast muscle, parallel to our observations.

Endogenous antioxidant systems comprise non-enzymatic lipophilic and hydrophilic compounds including HCDs to counteract the action of pro-oxidants in muscle tissues^47^. Higher carnosine in the Kadaknath meat prompted us to further explore the physiological properties in its meat as carnosine exhibits a dynamic list of health benefits due to its antioxidant, metal chelating, and anti-glycation abilities^40^. Complex composition and the presence of various antioxidants in heterogeneous foods such as meat make it difficult to decipher the role of each antioxidant. Hence, different in vitro assays have been introduced to screen their overall antioxidant activity^48,49^. These tests involve different chemical mechanisms and may address different aspect(s) of antioxidant properties. A recent publication Sehrawat et al.^50^ reported the superior antioxidant capacity of Kadaknath meat in comparison to the Cobb broiler based on free radical scavenging assays that involved single electron transfer (ET) reactions. As complementary assays provide more conclusive information on the antioxidant properties for a given sample the ORAC, a classic and new tool for measuring the antioxidant capacity of biomolecules using the hydrogen atom transfer (HAT)^51^ method was included (Table 2).

**Table 2:** Antioxidant capacity of chicken meat estimated by Oxygen radical absorbance capacity (ORAC) and other in vitro assays.

Antioxidant assay/genotypes	ORAC (µM TE/g of tissue)	FRAP (mM Fe^2+^/g of tissue)	CUPRAC (mM TE/g of tissue)	ABTS (% inhibition)	DPPH (% inhibition)	MCA (% inhibition)
**Breast**						
Cobb Broiler	748.56 ± 7.48^a^	15.24 ± 0.40^a^	9 ± 0.24^a^	43.78 ± 1.47^a^	70.56 ± 0.59^a^	53.63 ± 1.79^a^
Kadaknath	804.01 ± 9.37^b^	26.97 ± 0.37^b^	12.76 ± 0.35^b^	52.72 ± 1.42^b^	73.26 ± 0.70^b^	62.71 ± 0.99^b^
**Thigh**						
Cobb Broiler	762.82 ± 9.19^a^	19.20 ± 0.31^a^	7.15 ± 0.24^a^	29.62 ± 1.27^a^	63.46 ± 0.56^a^	75.07 ± 0.98^a^
Kadaknath	810.80 ± 6.29^b^	33.85 ± 0.47^b^	8.76 ± 0.22^b^	30.14 ± 1.00^a^	66.75 ± 0.55^b^	80.75 ± 0.95^b^

(Values are mean ± SEM (n = 20). Values with different superscripts within same column differ for P < 0.05.

*FRAP* Ferric ion reducing antioxidant power, *CUPRAC* Cupric reducing antioxidant power, *ABTS* 2,2'-azino-bis(3-ethylbenzothiazoline-6-sulfonic acid), *DPPH* 1,1-Diphenyl-2-picrylhydrazyl, *MCA* Metal chelation activity^50^).

The total antioxidant activity identified by six in vitro methods showed Kadaknath chicken meat to be a better source of dietary antioxidants. The only other report of better HCDs, as well as, antioxidant activity of the dark chicken meat in comparison to different chicken breeds comes from the SF^18^. Therefore the black chicken meat can be valued as a better dietary source of natural antioxidants. Antioxidants are indispensable in the human body and food systems as they play a critical role in reducing oxidative processes and harmful effects of reactive oxygen species^52^. This significant observation gains importance because chicken meat otherwise also is a better dietary source of natural antioxidants including HCDs than pork, fish, and goat meat^12^. Chicken meat is sensitive to oxidative changes, which negatively influence taste, smell, and meat preservation. Therefore, the greater antioxidant potential of black chicken meat has health benefits along with better oxidative stability of the meat.

A high concentration of advanced glycation end-products (AGEs) can initiate actions in the human body that lead to various disorders and their associated complications, such as Alzheimer’s disease, atherosclerosis, diabetes, kidney disease, and chronic heart failure^53,54^. It has been shown that extracts from some plants and animals, can inhibit the formation of AGEs through their strong antioxidant properties^55^. The comparative study between Kadaknath and Cobb broiler noted a positive influence of their meat extracts on the inhibition of AGEs, and the Kadaknath meat was a better antiglycation agent (Fig. 5). The high antiglycation potential of Kadaknath meat extract was likely to be related to the presence of additional carnosine, a very good inhibitor of AGEs formation both in vivo^56,57^ and in vitro^58^. Carnosine may react directly with methylglyoxal and sequester it or its amino group and the imidazole ring may bind to reactive dicarbonyl groups^59^ or proteins may become “carnosinylated” at carbonyl groups, and that these may protect them from degradation and/ or crosslinking.

Meat is a source of several functional molecules considered to be vital from a nutritional perspective and having significant physiological significance including the HCDs (carnosine and anserine), and amino acids metabolite- creatine^5^. These functional molecules as well as the building block amino acids of HCDs (histidine and β-alanine) are lacking in plants^9^. Thus, meat is the only current food-based means to provide them in the human diet. Availability of these three bioactive molecules through diet is possible due to their good stability during processing (cooking, fermentation, and drying) and storage^32^. Moreover, at the physiological range of pH values, carnosine, anserine, and creatine are water-soluble and chemically stable. In humans, skeletal muscle carnosine content has already been shown to increase by the availability of carnosine, either by dietary or supplementary sources^60^. Published reports on the crucial roles performed by HCDs on human health and nutrition are gradually increasing^5,61^. The advantages of taking HCDs have been principally emphasized in the old-aged humans as the content of carnosine and overall mass of skeletal muscle decrease with age^36^. Athletes commonly supplement their diet with carnosine to enhance performance^1^. Sufficient creatine in the diet is specifically emphasized for vegetarian athletes if they have fewer intakes of both creatine and its precursors (methionine, arginine, and glycine)^61^. Poultry has the higher content of total HCDs among farm animal species (turkey > chicken > horse > pig > rabbit > beef). Results suggest that Kadaknath meat may be considered as a potential dietary source of functional biomolecules for human beings. The information on the higher chicken HCD content is more relevant currently as carnosine has anti-viral properties^14^ and is a promising nutrient for prevention, and support during the COVID infection^1^. The data presented in this report will be useful for designing future research on the health benefits of black chicken meat.

## Conclusion

Indian Kadaknath chicken meat may be categorized as a functional food for optimizing human growth, development, and health as it is enriched in bioactive dipeptide carnosine and a nutritionally important source of physiologically significant nutrients anserine, and creatine. It has good antioxidant and antiglycation capacity. Information on better nutritional quality will enhance the research targeting the commercial potential of its meat in the fields of functional foods, cosmeceuticals, and nutraceuticals. This value addition can promote Kadaknath backyard poultry farming leading to women empowerment and socio-economic upliftment in rural India. Findings contribute towards enhancing public awareness on the health benefits of black chicken meat and further scientific investigations.

## Materials and methods

### Ethics statement

The study was approved by the Ethics Committee of the Veterinary College, Nanaji Deshmukh Veterinary Science University, Jabalpur vide order number 4040 dated 18.12.2018. No in vivo experiment was conducted. Birds were sacrificed and meat samples were collected as per the guidelines and regulations of the ethics committee in accordance with ARRIVE guidelines (https://arriveguidelines.org).

### Birds and sample preparation

Twenty male chickens each, of indigenous Kadaknath breed and commercial Cobb 400 broiler (Cobb) were reared at the poultry farm of College of Veterinary Sciences and Animal Husbandry, Jabalpur (23° 10′ 1.09" N 79° 57′ 0.22" E), India. The experimental design was approved by the Ethics Committee of the Veterinary College, Nanaji Deshmukh Veterinary Science University, Jabalpur (O No. 4040/ Dean/ Vety/ 2018 dated 18.12.2018). A deep litter system was followed for rearing the birds in the open-sided poultry house under identical conditions following standard and uniform management regimen. Birds were sacrificed at the age of marketing- 8 weeks for Cobb and 20 weeks for Kadaknath following standard scientific procedures as per the guidelines of the ethics committee. The breast and thigh meat were trimmed of visible fat and connective tissue, cut into small pieces and minced in a domestic meat blender (Panasonic MK-SW200, India) for 30 s. Minced samples were stored at − 80 °C in PE plastic bags wrapped with an aluminum sheet until use. Finely chopped breast and thigh meat samples of each bird were also preserved simultaneously in the RNALater^®^ (Ambion Inc., Austin, USA) filled cryovials. It was then stored overnight at 4 °C followed by storage at − 80 °C till further processing for RNA isolation.

For the preparation of meat extract, 2 g meat was homogenized in 20 mL of phosphate-buffered saline (PBS, pH 7.4) in an ice bath using a homogenizer (Benchmark Scientific D1000, USA). The homogenate was extracted in dark at 4 °C for 20 min followed by centrifugation at 10,000×*g* for 15 min at 4 °C. The solid residue was discarded and the aliquots of supernatant were stored at − 20 °C till further use.

### Measurement of HCDs (carnosine and anserine) and creatine contents

The quantity of di-peptides (anserine and carnosine) and creatine were determined in both the breast and the thigh tissue of the Kadaknath (n = 20) and Cobb (n = 20) following the slightly modified method of Mora et al.^62^. Briefly, 0.5 g chicken meat (breast or thigh) was homogenized (3000×*g*) with 0.1 N HCl (3 mL) for 1 min. The supernatant obtained after centrifugation at 10,000*g* for 20 min at 4 °C was filtered through Whatman No. 4 filter paper. Deproteinization of the above supernatant (250 µL) was achieved by mixing it with acetonitrile (750 µL) and leaving it undisturbed for 20 min at 4 °C. Next, the mixture was centrifuged for 10 min (10,000×*g*) at 4 °C and filtered through a 0.22 µm membrane filter (Millipore, Sigma, St. Louis, MO, USA) to obtain the sample for analysis using high-performance liquid chromatography (HPLC). Twenty microliters of each sample were injected into an HPLC system (1260 Infinity; Agilent Technologies, USA) equipped with the Zic-HILIC silica column (4.6 × 150 mm, 3 μm; Waters, Milford, MA, USA). The column temperature was kept at 35 °C. The mobile phases consisted of solvent A (pH 7, 0.65 mM ammonium acetate in water:acetonitrile, 25:75, v/v) and solvent B (pH 6.8, 4.55 mM ammonium acetate in water:acetonitrile, 70:30, v/v). The flow rate was 1.2 mL/min for 8 min with a linear gradient (0% to 100%) from solvent A to B. A diode array detector was used at 214 nm to measure HCDs and creatine contents. Standard curves for carnosine, anserine, and creatine were drawn using the respective standards (Sigma-Aldrich, St. Louis, MO, USA). A regression equation was obtained using the area under the curve (AUC) of generated peaks. Carnosine, anserine, and creatine content was quantified by plotting the AUC of each sample against its standard curve data and reported as mg/ g of wet tissue weight.

### Relative mRNA abundance analyses using quantitative real-time polymerase chain reaction (qRT-PCR)

Total RNA was extracted from the breast tissue of Kadaknath (n = 20) and Cobb (n = 20), and thigh tissue of Kadaknath chicken (n = 20) using TriReagent (Sigma-Aldrich). Column purification of the isolated RNA was done with the Qiagen RNeasy kit (Cat. No. 74004) following the manufacturer’s instructions. RNA concentration and quality were estimated using a Nanodrop ND-1000 spectrophotometer (Thermo, Scientific, Waltham, MA). The samples were considered for downstream analysis if A260/A280 and A260/A230 ratios were not less than 2.0.

Purified RNA from each sample (2 μg) was reverse transcribed to the cDNA with the SuperScript® III First-Strand Synthesis System (Catalog number: 18080051; ThermoFisher Scientific) following the manufacturer’s instructions. SYBR Green I chemistry was used to estimate the differential expression of genes related to the carnosine accumulation^43^ namely CARNS1 (Carnosine synthase 1), CNDP1 (Carnosine dipeptidase 1) and (Carnosine dipeptidase 2), and β-alanine transporters—SLC6A6 (Solute carrier family 6, member 6), and SLC6A14 (Solute carrier family 6, member 14), and SLC36A1 (Solute carrier family 36, member 1) on real-time PCR system (LightCycler® 480 Instrument II, Roche Life Science, Germany). Primers (Table 3) were synthesized from Integrated DNA Technologies, USA. Four replicates were performed for each sample and amplifications were performed in triplicates in a 10 μL reaction volume containing 2 μL of the cDNA, 5 μL of 2X Power SYBR® Green Reagent (Applied Biosystems, Life Technologies), 0.3 μL each of forward and reverse primer (concentrations between 150 and 300 nM), and nuclease-free water to adjust the volume at 10 μL. Cycling conditions were 5 min at 95 °C, followed by 45 cycles of 10 s at 95 °C, 30 s at 60 °C, and 10 s at 72 °C. To confirm the specificity of all individual amplification reactions, a dissociation curve analysis was included at the end of the amplification: 95 °C for 5 s, 60 °C for 1 min, 95 °C for 15 s and 60 °C for 1 min. One single peak of the target gene quantitative melting curve indicated the absence of nonspecific amplification and primer dimer formation in the amplification process. β actin was used as the reference gene and the relative expression of each gene was quantified (fold change) using the 2^−ΔΔCt^ method^63^.

**Table 3:** Characteristics of the primers used in quantitative RT-PCR analysis of Cobb 400 and Kadaknath chicken meat.

Role	Gene	Gene name	Primer sequence F, forward (5´ to 3´) R, reverse (3´ to 5´)	Annealing temperature	Product size (bp)	NCBI Gene ID
Carnosine synthesis	CARNS1	Carnosine synthase	F: CTGCCCTGGAAGAATTTGTG	60 °C	172	100,359,387
			R: GACAGCAACCAGCGAGAGAG			
Carnosine hydrolysis	CNDP1	Carnosinase-1	F: ATTCTCCATTCGCCAAGTTC		NA	421,012
			R: GCATCTGCATCACCAATAGG			
	CNDP2	Carnosinase-2	F: AAACCTTGGGTGTCAGACTT		149	421,013
			R: ACATTCTTGCCTGTTGCTTC			
β-alanine transport	SLC6A6	Solute carrier family 6, member 6	F: GGGAAATCTTCATCGCTAT		169	416,041
			R: CCATAAACCCAGGCTAC			
	SLC36A1	Solute carrier family 36, member 1	F: CACGGCAGTTCCCTCTGAT		135	770,250
			R: AGCAGTTGGGCAGGTTGAG			
	SLC6A14	Solute carrier family 6, member 14	F: AAACACGCCTCGTAAATGA		NA	100,857,521
			R: CACGATGTTGCCAGTCTCA			
Reference gene	ACTB	β-actin	F: ATCCGGACCCTCCATTGTC		120	396,526
			R: AGCCATGCCAATCTCGTCTT			

*NA* Not amplified.

### Antiglycation capacity estimation by measurement of advanced glycation end products (AGEs)

In vitro antiglycation capacity of the breast and thigh meat of Kadaknath chicken (n = 20) was compared with the corresponding activity in Cobb broiler (n = 20) by testing the ability of the extracts to inhibit the methylglyoxal mediated development of bovine serum albumin (BSA) fluorescence. The method of Abdelkader et al.^64^ was followed, with slight modifications. BSA and methylglyoxal (Sigma Aldrich, USA) were dissolved in phosphate buffer (50 mM, pH 7.4) to a concentration of 50 mg/mL and 3 mM, respectively. An equal volume (500 μL) of methylglyoxal solution and tissue extract prepared in the same phosphate buffer was mixed in 10 mL screw-capped glass tubes with 0.02% Sodium azide, serving as an antimicrobial agent. The mixture was incubated for 2 h at 37 °C. Bovine serum albumin (BSA) (500 μL; 50 mg/mL) was added to each tube and the mixture was again incubated at 37 °C for 72 h in darkness. The reaction was terminated by adding 50 µL of 100% (w/v) trichloroacetic acid (TCA) followed by centrifugation (12,000×*g*) for 4 min at 4 °C. The pellet was washed with 50 µL of chilled TCA (5%). The pellet containing AGEs was dissolved in 100 μL PBS. The plates were examined for the development of specific fluorescence at 370, and 440 nm (excitation and emission, respectively) using the microplate reader (Infinite F200 Pro, Tecan Austria GmbH, Austria). Phosphate buffer was used as a blank. Aminoguanidine (30 mM) and carnosine (10 mM) were used as the standard glycating agents. Triplicate samples were run for each set, and the percent inhibition of AGEs formation by meat extracts was calculated using the following equation (FI: fluorescence intensity).

% Inhibition = [1 − (FI of extract/ FI of control)] × 100.

### Antioxidant activity

Oxygen radical absorbance capacity-fluorescein (ORAC) assay was carried out using the ab233473 ORAC assay kit (Abcam, UK) following the manufacturer’s instructions. Briefly, 25 μL of the meat extract was added to the different wells of 96-well microplate. Fluorescein solution (150 μL, 1X) was added to each well, thoroughly mixed and the plate was incubated for 30 min at 37 °C. After incubation 25 μL of the free radical initiator solution was added, thoroughly mixed and the microplate was immediately placed in the microplate reader (Model: Infinite F200 Pro, Tecan Austria GmbH, Austria). The decay in fluorescence was recorded every minute for 60 min with an excitation wavelength of 300 nm and emission at 380 nm. A blank using phosphate buffer instead of the extract and seven dilutions of Trolox (6-hydroxy-2,5,7,8–tetramethylchroman-2-carboxylic acid) as the antioxidant standard (2.5–50 µM) were also carried out in each assay. Three independent assays were performed for each sample. The area under the curve (AUC) for each sample and standard was calculated using the final assay values and the linear regression:

$$\frac{AUC }{RFU0}=1+\frac{RFU1}{RFU0}+\frac{RFU2}{RFU0}+\frac{RFU3}{RFU0}+\cdots +\frac{RFU59}{RFU0}+\frac{RFU60}{RFU0}$$

where: *RFU0* = Relative fluorescence value of time point zero, *RFUx* = Relative Fluorescence value of time (minutes) points. The net AUC was obtained by subtracting the AUC of the blank from the AUC of each sample and standard as:

$${\text{Net AUC}}={\rm AUC}\left({\rm Antioxidant} \right)-{\rm AUC}\left({\rm Blank} \right).$$

The Trolox standard curve was prepared by plotting the net AUC on (Y-axis) against the concentration on X-axis. The regression equation between net AUC and antioxidant concentration was calculated. The slope of the equation was used to calculate the µM Trolox Equivalents (TE) of the unknown sample (ORAC value) expressed as µM TE/ g tissue, the Trolox equivalent antioxidant capacity (TEAC).

### Statistical analyses

Data analyses for the quantification of carnosine, anserine, and creatine and the measurement of antioxidant and antiglycation potential were performed by Statistical Package for Social Sciences (SPSS version, 10.0, SPSS Inc., Chicago, IL, USA). The results of triplicate independent measurements were analyzed using one-way analysis of variance and mean comparisons were conducted using Duncans’ post hoc or *t* test. Statistical significance was set at 95% confidence level (P < 0.05). Values were shown as means and standard error (SE). mRNA expression of enzymes and transporters related to carnosine accumulation inside the cell was evaluated in breast and thigh tissue using two-way ANOVA followed by Bonferronis’ post hoc test using the Graphpad Prism 8.0 software package (https://www.graphpad.com/scientific-software/prism/).

## Data Availability

The datasets generated during and/or analyzed during the current study are available from the corresponding author on logical request.
